# Topical Delivery of Senicapoc Nanoliposomal Formulation for Ocular Surface Treatments

**DOI:** 10.3390/ijms19102977

**Published:** 2018-09-29

**Authors:** Jie Liang Phua, Aihua Hou, Yuan Siang Lui, Tanima Bose, George Kanianthara Chandy, Louis Tong, Subbu Venkatraman, Yingying Huang

**Affiliations:** 1 School of Materials Science and Engineering, Nanyang Technological University, Nanyang Avenue, Singapore 639798, Singapore; phuajl@ntu.edu.sg (J.L.P.); yslui@ntu.edu.sg (Y.S.L.); 2 Singapore Eye Research Institute, Singapore 169856, Singapore; hou.aihua@seri.com.sg (A.H.); louis.tong.h.t@singhealth.com.sg (L.T.); 3 Duke-NUS Medical School, Singapore 169856, Singapore; 4 Lee Kong Chian School of Medicine, Nanyang Technological University, Singapore 308232, Singapore; tanimabose@gmail.com (T.B.); gchandy@ntu.edu.sg (G.K.C.); 5 Singapore National Eye Center, Singapore 168751, Singapore; 6 Yong Loo Lin School of Medicine, National University of Singapore, Singapore 117597, Singapore

**Keywords:** liposomes, senicapoc, ocular, hydrogel

## Abstract

Topical ophthalmologic treatments have been facing great challenges with main limitations of low drug bioavailability, due to highly integrative defense mechanisms of the eye. This study rationally devised strategies to increase drug bioavailability by increasing ocular surface residence time of drug-loaded nanoliposomes dispersed within thermo-sensitive hydrogels (Pluronic F-127). Alternatively, we utilized sub-conjunctival injections as a depot technique to localize nanoliposomes. Senicapoc was encapsulated and sustainably released from free nanoliposomes and hydrogels formulations in vitro. Residence time increased up to 12-fold (60 min) with 24% hydrogel formulations, as compared to 5 min for free liposomes, which was observed in the eyes of Sprague-Dawley rats using fluorescence measurements. Pharmacokinetic results obtained from flushed tears, also showed that the hydrogels had greater drug retention capabilities to that of topical viscous solutions for up to 60 min. Senicapoc also remained quantifiable within sub-conjunctival tissues for up to 24 h post-injection.

## 1. Introduction

Revolutionary approaches to drug delivery systems in the treatment of ocular diseases have rapidly emerged over the past decades. In general, the treatment of ocular surface disease relies on topical administration of drugs. In contrast, the treatment of intraocular disease largely depends on diffusion of drugs across the cornea to reach the anterior and posterior segments of the eye. Topical eye drops, though convenient and with high patient compliance, suffer low bioavailability due to washouts by static and dynamic defense mechanisms of the eye. Less than 5% of topically administered drugs reach the anterior segment and an even smaller fraction reaches the posterior chamber [1]. This suggests that dynamic clearance of drugs occurs primarily via tears or at the ocular surface. Treatment of ocular surface diseases have also faced many challenges over the decades either due to poor treatment efficacies of topical eye drops or poor acceptability of the patients due to more invasive routes of administration [1,2,3]. Commercially available free drug topical eye drops are typically prepared in high concentrations to achieve therapeutic dosages [4], but they still require frequent administration, and in some occasions induce toxicity due to fluctuating doses of drug [5]. Other common problems include drug spiking dosages and efficacy crashes [1].

New drug delivery strategies have been developed to prolong the bioavailability of pharmaceutical drugs at the ocular surface. These include devices such as the corneal shield [2,6], contact lenses [3,7,8,9], nano-particulate systems [10,11,12,13,14,15,16,17,18], and in situ hydrogels [19,20,21,22,23]. Corneal shield and drug eluting contact lenses were developed to provide more controlled and sustained release of drugs when in contact with the tear fluid. Nano-particulates and vesicular systems were primarily developed to overcome challenges related to poor solubility of non-polar drugs, duration of drug delivery [10,11,16,17,18], as well as targeting to specific sites [12,15].

For example, corneal shields are made of collagen derived from porcine or bovine origins and are synthetically manufactured to undergo dissolution after a specific amount of time [6]. Collagen being a major component of physiological systems is biocompatible and bioresorbable, making it a suitable bio-device for drug delivery and wound healing. Corneal shields absorb tear fluids upon application into the eye, softening into a pliable film that conforms to the ocular surface resulting in optimal surface interaction with sustained drug release profiles based on largely bulk dissolution and diffusion [1]. Further developments by crosslinking were made to corneal shields to increase the dissolution time, hence allowing the bio-device to act as a depot for sustained release via diffusion [2]. However, a major drawback of commercially available corneal shields lies in its degree of opacity as compared to contact lenses [2]. Unlike corneal shields, contact lenses are cross-linked transparent hydrogels that do not undergo dissolution. Made of biocompatible polymers (e.g., poly (methyl methacrylate) (PMMA), hydroxyethylmethacrylic acid (HEMA), silicone), drug-eluting contact lenses are hydrophilic hydrogels that absorb water and swell for the exchange of small polar molecules, which facilitates the sustained release of drugs by diffusion [3,7]. To enable non-polar drugs to be loaded into gels, they could be first encapsulated within micro emulsions [7], liposomes [8], and even nanoparticles [9] prior to suspension within contact lens matrices [3]. Although contact lenses have been well accepted for the purpose of corrective vision, they have drawbacks due to deprivation of epithelial oxygen density to the cornea, reducing tear film stability, and potentially increasing the risk of ocular surface microbial infections.

In situ gel formulations for pharmaceutical applications has also garner its fair share of attention over the past decade. These formulations are typically synthesized to undergo aqueous-based sol-gel transition above physiological temperatures to form physically cross-linked gel-like matrices. Temperature transitions of these formulations in aqueous solutions are related to the miscibility gap in their respective temperature-composition diagram which corresponds to either a lower critical solution temperature (LCST) [19,20,21,22,23] or upper critical solution temperature (UCST). Such formulations are also particularly of interest to ocular surface treatments since they can be administered as eye drops, and after instillation, can form physically cross-linked depots within the eye to increase residence time and hence therapeutic bioavailability [21,22]. Some commonly known thermo-sensitive polymers used for biological applications are polyxamers, poly-(*N*-isopropylacrylamide), poly-(vinyl alcohol), and methylcellulose. Co-polymers consisting of thermo-sensitive and pH sensitive polymers (e.g., Carbopol) are also often added in combination to increase gel strength and achieve suitable consistencies in texture [23]. In the gel state, in situ hydrogels swell in the presence of fluid, resulting in extended retention time, hence improving drug bioavailability.

As a delivery system for the ocular surface, liposomes have several advantages. They are biocompatible [15,24] because they are typically made of phospholipids resembling mammalian cell membranes. Protective mammalian surfactants that are secreted at air-liquid interfaces line the pulmonary tract and the ocular surface, as well many other parts of the body to providing lubrication. Dipalmitoylphosphatidylcholine (DPPC), more commonly known as lecithin, is a major lipid component found in these surfactants. They are fully biodegradable [14,17,24], relatively non-toxic, and promote intracellular uptake [17]. It has been reported that conjugation with hydrophilic polyethylene glycol can help to reduce uptake by macrophages [15,16,25]. Apart from surface modifications, it has also been reported that the increase in physiological circulations time has also been attributed greatly to the decreasing in liposomal size, which is resulted from the enhanced evasion of liposomes from the mononuclear phagocytic system (MPS) [16,26]. A liposome is made up of an aqueous core where hydrophilic drugs can be encapsulated, and surrounded by a non-polar bilayer, where hydrophobic drugs can be associated, making it possible to incorporate multiple payloads within each vesicle. Liposomes can also be easily manipulated to achieve monodispersity from a range of various sizes between 80 nm to 10 µm [15]. Nanoliposomes with size below 100 nm exhibit reduced light scattering and provides adequate transparency, making it suitable for ocular applications [18]. Although drug concentrations have been reported in both anterior and posterior segments of the eye after sub-conjunctiva administration [4,5,10,27], however these finding were not a representation of potential treatments of ocular surface disease, apart from one other article that describes sub-conjunctival injections of nanoliposomes as a potential treatment of diseases that targets the ocular surface [28].

To maintain high patient compliance while achieving therapeutic efficacy, we propose the use of drug-loaded nanoliposomes dispersed within thermo-sensitive hydrogels (Pluronic F-127), via a topically applicable hydrogel formulation or a minimally invasive sub-conjunctival injection to increase the residence time and bioavailability of ocular therapeutics. Here, we also describe the encapsulation of a specific inhibitor of the calcium-activated potassium channel, K_Ca_3.1, for delivery to the ocular surface. K_Ca_3.1 channels are validated targets for immunomodulation and in reducing conjunctiva and corneal [29] as well as other TGF-β induced fibrosis [29,30]. K_Ca_3.1 inhibitors are reported to be effective in the treatment of corneal alkali burn in a mouse model [31]. Senicapoc, the drug chosen for our study, has also been shown to be biologically safe during its advancements in human trials [32,33]. Pluronic F-127 is a polymer that displays thermo-sensitive gelation and is concentration dependent. It portrays a lower critical solution temperature dependence that is only exhibited with a minimum of 16 wt% solids at a fusion point on the phase diagram [34]. Its biocompatibility [35,36], composition variability, low toxicity [37], and spontaneous formation of transparent hydrogels at physiological temperatures makes it a suitable choice of ophthalmic applications [23,35]. Pluronic F-127 hydrogels has also been reported to form matrixes that stabiles liposomes [38], as well as reduce drug degradation [39], hence enhancing bioavailability. In this study, we prepared Senicapoc-loaded liposomes within a thermo-sensitive hydrogel matrix of different concentrations of Pluronic F-127. Our primary objective was to prolong the release, and increase the bioavailability of Senicapoc within the ocular surface following patient compliant topical administration of hydrogel liposomal formulations. In addition to eye drops, we also considered an alternative use of sub-conjunctival injection, which potentially serves to bypass the tight junctions of the corneal epithelium [40,41]. The widely known inefficiency of topical therapeutics attributed to challenges of low penetrability of the corneal epithelium can be overcome by sub-conjunctiva injections which serve as an alternative yet independent route of trans-sclera penetration [4,5] and to prolong the potential treatment of ocular surface disease [4,5,28,42].

## 2. Results

### 2.1. In Vitro Size and Drug Loading Stability of Senicapoc-Loaded Liposomes

**Figure 1:**
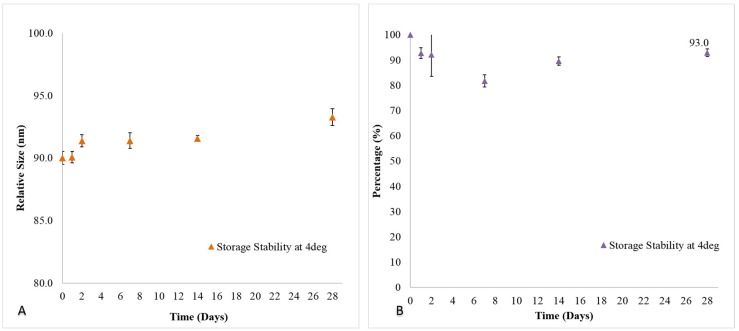
(**A**) Size and (**B**) drug loading stability of Senicapoc-loaded liposomal formulation at 4 °C storage conditions over a period of 28 days. All data are reported as mean ± SD of triplicates.

**Figure 2:**
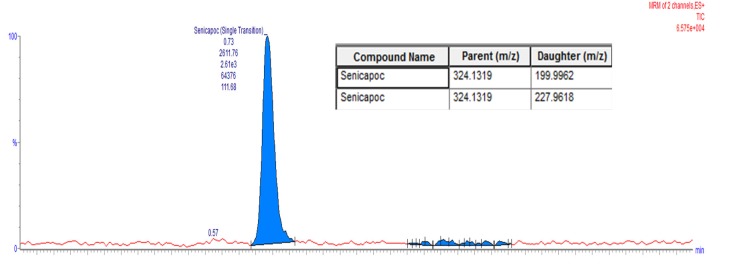
Liquid Chromatography Mass Spectroscopy (LC-MS) Chromatogram of Senicapoc.

In this study, we monitored the liposome size and drug loading (formulation stability) at storage condition of 4 °C to determine its potential shelf-life over a period of 28 days (Figure 1). At endpoint, it was observed that 20 mM Senicapoc-loaded DPPC liposomes had no significant changes in its size with an average of 91.3 ± 1.2 nm (Figure 2) over a period of 28 days as compared to the initial fabricated size of 90.0 ± 0.5 nm (*n* = 3). Extruded Senicapoc-loaded Liposomes were fabricated consistently with an initial drug loading of 7.73 ± 0.23 mol % (*n* = 3). The liposomal formulation maintained a drug loading capacity of 93.0 ± 1.5% of the initial drug content after a period of 28 day storage at 4 °C.

### 2.2. In Vitro Release of Senicapoc-Loaded Liposomes

**Figure 3:**
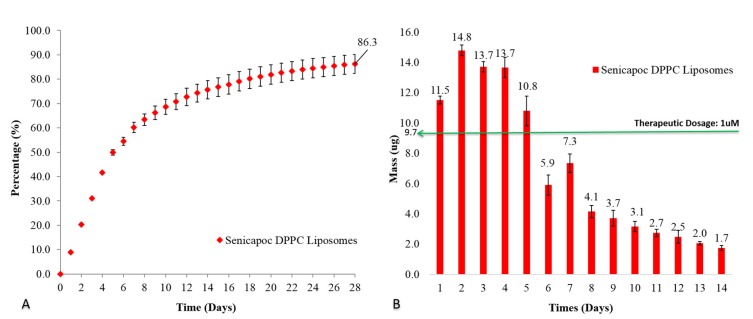
(**A**) Cumulative percentage release and (**B**) daily mass release of Senicapoc from drug-loaded liposomal formulation over a period of 28 days at 37 °C. All data are reported as mean ± SD of duplicates.

The cumulative drug release of Senicapoc from DPPC liposomes (Figure 3) was observed over a period of 28 days before achieving a cumulative released amount of 86.3 ± 3.9%. Therapeutic dosages of 1 µM of Senicapoc, established for the suppression of 90% naive and central memory T cells [43,44,45,46,47,48] had been sustainably achieved initially within 5 days, and possibly up to 7 days before an additional instillation was required.

### 2.3. In Vitro Release of Senicapoc-Loaded Liposomal Hydrogel Formulation

**Figure 4:**
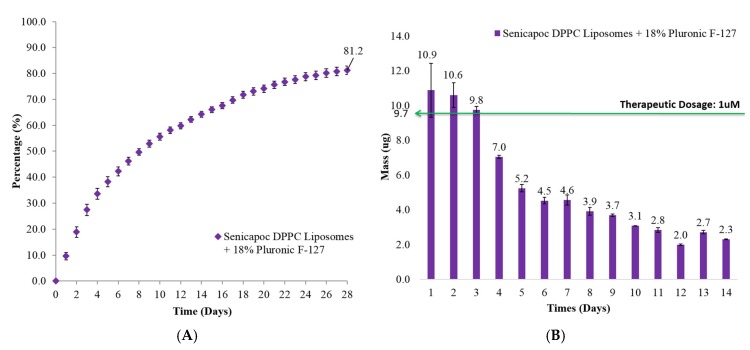
(**A**) Cumulative percentage release and (**B**) daily mass release of Senicapoc from drug-loaded liposomes dispersed in 18% hydrogel formulation over a period of 28 days at 37 °C. All data are reported as mean ± SD of duplicates.

The release of Senicapoc from the Senicapoc-loaded liposomal dispersion within an 18% hydrogel (Figure 4) also exhibited a sustained release profile over a period of 28 days before achieving a cumulative release of 81.2 ± 1.7%. For the first day, Senicapoc mass release was not significantly different (*p* = 0.67) from that of the liposomal hydrogel dispersion formulation as compared to the free liposomal formulation (Figure 3). Subsequent release from day 2 to day 4 and day 13 (*p* < 0.05) were significantly lower, while days 5 and 7 (0.05 < *p* < 0.07) and day 6 and 14 (*p* = 0.19 and *p* = 0.13 respectively) as well as the rest of the time points (*p* > 0.2), however showed no significant difference between the liposomal hydrogel dispersion formulation as compared to the formulation without the hydrogel matrix.

### 2.4. In Vivo Residence of Hydrogel on Ocular Surface of Sprague Dawley Rats

**Figure 5:**
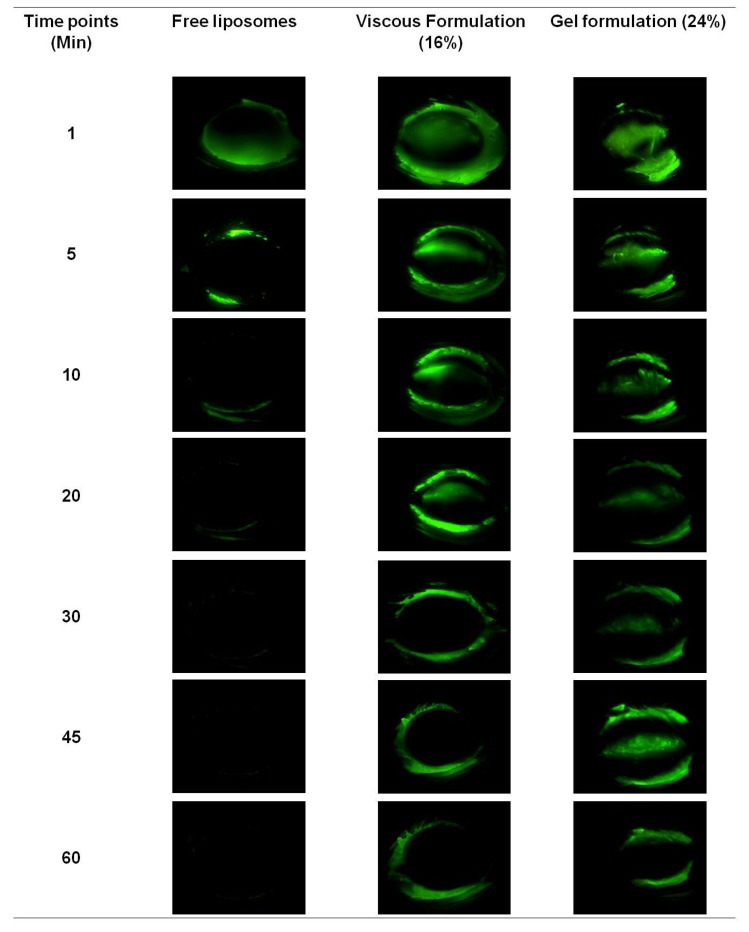
Residence images (Micron IV Imaging) of free fluorescein-tagged liposomes and hydrogel formulations in eyes of anesthetized Sprague-Dawley rats. Two rats were used for each formulation.

The fluorescein-tagged free liposomes, viscous formulation and gel formulation were applied onto eyes of anesthetized rats respectively to determine the retention time on the ocular surface. To compensate for the dilution of hydrogel formulation by tears present in the rat eyes, 24% hydrogel formulations were prepared instead of 18% hydrogel formulations. This consideration was to prevent the excessive dilution of the gel formulation to drop below the critical solution composition for gelation at physiological temperature of 37 °C. Fluorescein signals from these formulations were recorded by the Micron IV imaging system under cobalt blue filter at different time points (Figure 5). Since cobalt blue light lacks the intensity to generate auto-fluorescence from eye tissues, and in the absence of fluorescein, all baseline images taken appeared black with no difference between the rats (not shown in Figure 5).

As seen in Figure 5, the free liposomes could not be observed on rat ocular surfaces 5 min after application, whereas the viscous formulation could be observed for up to 30 min, and the observed retention of gel formulation was the longest at 60 min. During eye blinking, free liposome and viscous formulations spread more rapidly on the ocular surface than the gel formulation, and thus were cleared faster. The blinking of the rat’s eyes had a tendency to expel the excessive formulation, which explained the observed fluorescence on the eyelids and the eyelashes for all formulations.

The retention time of viscous formulation and gel formulation on ocular surface of anesthetized rat was also examined by fluorophotometry. As the scanning software of Fluorotron Master™ was designed according to the human eye, the actual magnitude of the measurements for the rat eye distances in the horizontal axis was not applicable for this purpose, but this did not affect the interpretation of fluorescence signals in the vertical axis.

**Figure 6:**
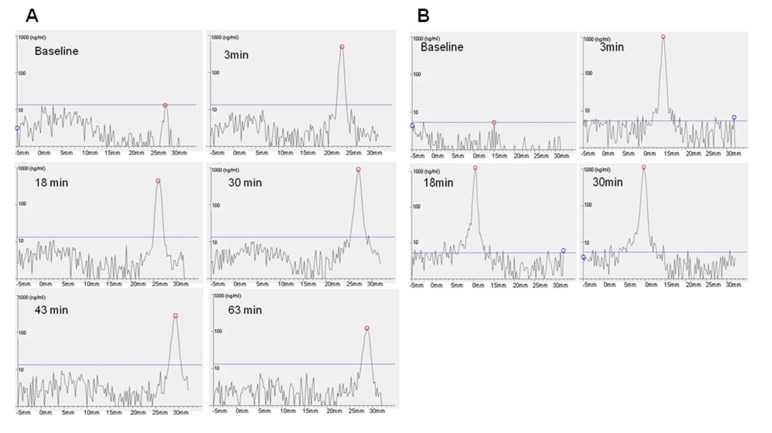
Fluorotron master scan of fluorescein-tagged liposomes migration from (**A**) viscous formulation and (**B**) gel formulation within the eyes of anesthetized Sprague-Dawley rats. Two eyes of one rat were used for each formulation.

Although the clearance of foreign substances from ocular surface was dominant (>75% [1]) mainly through eye blinking and the nasolacrimal tear drainage system [4,5,10,14,28], however, the consideration for drug penetration was not neglected. In Figure 6, the fluorescence signal of the viscous formulation could be observed at the surface of the eye even after 60 min, in the absence of blinking. It is also interesting to note that the fluorescence peak for gel formulation shifted posteriorly towards the back of the eye within the initial 3 min after instillation and eventually residing within the eye for up to 30 min in the study. Fluorescein has been widely used as a fluorophore for labeling to study the distribution of liposomal vesicles biologically [49,50]. It is also commonly known for its ease of photo-bleaching. However, in this study, caution was taken to ensure that exposure time of all fluorescein-labelled formulations after applications were kept constant and away from excessive photo-excitation as a result of light exposure, hence eliminating inconsistent photo-bleaching of formulations during measurements. Furthermore, as the hydrophilic carboxyfluorescein is chemically attached to the hydrophilic head of the lipid as used in this study, the labelled liposomes that were subsequently fabricated remained amphiphatic. Lee J. et al. [49] also reported that carboxyfluorescein-conjugated PEGylated DMPC-based liposomes exhibited increased penetration of the retina layers as compared to the same liposomes with carboxyfluorescin loaded in the aqueous core, which did not show any sign of facilitated penetration. Therefore, the carboxyfluorescein- labelled liposomal formulation described in this study may have been likely to exert an influence on the penetration of the ocular surface as shown in Figure 6B. Importantly, the penetration of the fluorescence-labelled liposomes can be attributed to the extended residence of the gel formulation at the ocular surface (Figure 5), which was comparably not observed for the viscous formulation.

### 2.5. In Vivo Pharmacokinetic Analysis of Eye-Flush Tears

**Figure 7:**
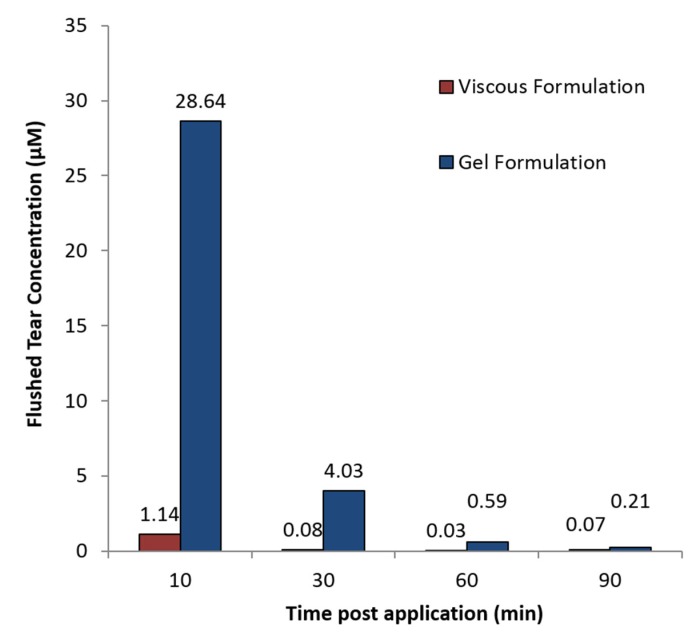
Senicapoc concentration in flushed tears after topical administration of hydrogel formulations in anesthetized Sprague-Dawley rats.

The topical instillation of 10 µL Senicapoc-loaded liposomal hydrogel formulations into the eyes of Sprague-Dawley rats were well-tolerated with no significant sign of irritation or redness being observed. Eye-flush tears were collected and analyzed as described in the materials and method section. Figure 7 shows the higher concentrations of Senicapoc were present in the eye at all-time points consistent with increased residence time provided by the 24% gel formulation (shown previously in Figure 5). This has implications for improvement in therapeutic bioavailability of the drug compared to drug associated with free liposomal eye drops.

### 2.6. In Vivo Pharmacokinetic Analysis of Sub-Conjunctival Injection

**Figure 8:**
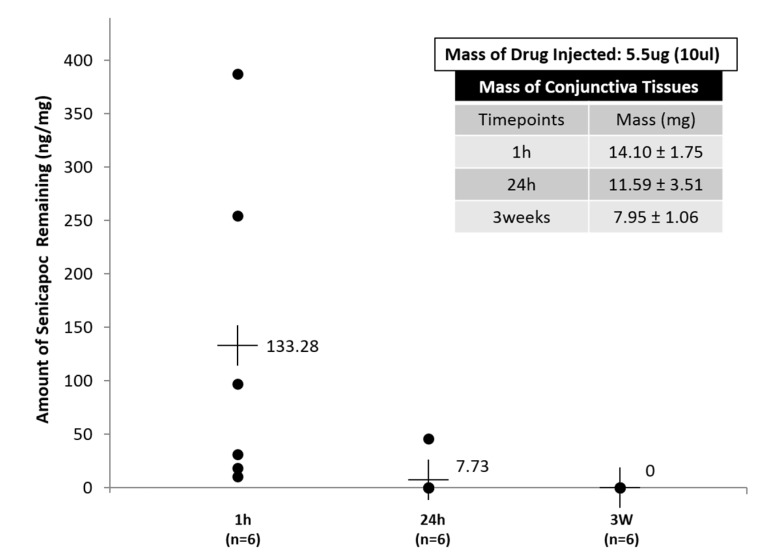
Residual Senicapoc concentration in sub-conjunctiva tissues at 3 different time-points (1 h, 24 h and 3 weeks). All data are reported as mean ± SD of *n* = 6.

The delivery of nanoliposomes without gels via sub-conjunctiva injection was investigated as an alternative measure to increase bioavailability to the ocular surface. It was found that Senicapoc from the Senicapoc-loaded DPPC liposomes in the sub-conjunctiva tissues were detectable and quantifiable up to 24 h (Figure 8), indicating a rapid but sustained delivery of Senicapoc, associated with a rapid clearance rate. At 3-week time-point, the presence of Senicapoc was no longer detectable, and was representative of a complete liberation from the injection site. This method of quantification performed correlated indirectly to the combination of drug release kinetics and the migration of liposomes away from the injection depot, and were in alignment with the experimental findings of Natarajan et al. [51]. In another study performed by Natarajan et al., they also displayed pre-clinical efficacy of latanoprost in glaucoma treatment (i.e., anterior diseases) beyond 90 days, after sub-conjunctiva injection of latanoprost-loaded liposomes into rabbit eyes [52]. This further indicates the potential residence and bioavailability of the therapeutic and the drug-loaded liposomes in the eye after their migration away from the injection site.

Several distributional pathways of the nanoparticles have been identified within the eye, including nasolacrimal and lymphatic clearance routes via lymph nodes [28]. Feng et al. [28] found that the internalization and distribution of their pRNA nanoparticles had different terminal locations (conjunctiva, cornea, sclera, and retina), related to the shape and size of the nanoparticles, and encouragingly, nanoparticles remained detectable by fluorescence up to 20 h. Another study that used sub-conjunctiva injection of poly(lactic-co-glycolic acid) (PLGA) nanoparticles containing brinzolamine for glaucoma treatment showed positive efficacy data in the reduction of intraocular pressure of up to 7 days after a single injection, which was attributed to the longer mean retention time of the PLGA nanoparticles compared to eye drops [27]. Collectively, these data highly suggest that minimally invasive sub-conjunctiva injections could reduce the distributional clearance of Senicapoc-loaded liposomes within the eye, and could serve as depots for sustained release in treatment of ocular diseases.

## 3. Discussion

In this study, we focus on nanoliposomal delivery systems for the treatment of ocular surface diseases. We explored a novel engineered Senicapoc-loaded topical liposomal hydrogel eye drops as a strategy to prolong corneal treatment efficacies, while we also explored the use of sub-conjunctiva injection as an indirect treatment method to target ocular surface diseases. Senicapoc-loaded liposomes were prepared in a thermo-sensitive hydrogel matrix of different concentrations of Pluronic F-127. In vitro results show that Senicapoc can be released sustainably from DPPC liposomes regardless of whether the liposomes are free or dispersed within a Pluronic F-127 hydrogel over an extended period of 28 days. In vivo studies show that Pluronic F-127 hydrogel at 24 wt% concentration increases the residence time of the nanoliposomes on the surface of the eye, also increases bioavailability and supports the penetration of the nanoliposomes into the eye.

The greatest challenges faced by topical eye drops is commonly attributed to the corneal route of penetration [4]. Since it is also widely reported that topical solutions are highly ineffective in travelling to the posterior segment of the eye [4,10], the dominant residence of hydrogel formulation which improves bioavailability of the therapeutic at the ocular surface and the anterior chamber. It is an encouraging opportunity for the use in ocular surface treatments.

In vitro experiments showed that free liposomal formulations achieved therapeutic dosages for the initial 5 days while liposomal hydrogel formulation exhibited the same for its initial 3 days. The increase in the viscosity of the formulation is attributed to the presence of physically cross-linked polymer of Pluronic F-127 [35]. That could contribute to the slight deduction of initial release of Senicapoc from hydrogel formulation. In vivo experiments shows the liposomal hydrogel formulation increases residence time by up to 6-fold (viscous solution) and 12-fold (hydrogel) compared to free liposomal formulation (Figure 5). Being a physically cross-linked hydrogel, Pluronic F-127 will eventually be eliminated through the nasolacrimal pump as a results of the breakdown of the hydrogel by a mixture of dilution by tear replenishment, erosion and degradation mechanism [37]. Furthermore, although it shows a higher daily release amount from the liposomes group, the clearance of unprotected liposomes can rapidly decrease the bioavailability of Senicapoc within the eye, as shown in Figure 5. While the slightly lower daily release of Senicapoc from the hydrogel formulation, the residence of the formulation is indeed prolonged much longer (up to 12 folds) based on the study performed in Figure 5. Hence, the rapid clearance experienced from the liposomal formulation is more likely to provide less bioavailability as compared to the hydrogel formulation, and the tendency for frequent administration is more likely to be required for the free liposomal formulation instead. The extended residence of the hydrogel formulation also delays clearance rates of the formulation at the ocular surface, hence providing sufficient time for penetration of the liposomes into the eye as seen in the results reported in Figure 6. This concept of prolonging the residence time on the ocular surface to increase penetrable bioavailability can further be justified by literature, where it was reported that use of Dexamethasone eye drops containing gamma-cyclodextrin-based nanogels may provide extended time for penetration, as well as providing a sustained release of dexamethasone while avoiding drug spikes [13]. Hence, these suggest a need to compromise between achieving a high initial therapeutic dosage and a prolonged retention of drug, which was further shown with a pharmacokinetic study using flushed tears (Figure 7). In a similar study performed by Hsiue et al. [21], drug and drug-loaded nanoparticles entrapped within a hydrogel matrix of thermosensitive poly-*N*-isopropylacrylamide exhibited prolong sustained effect compared to traditional ophthalmic eye drops in rabbits.

Due to static/dynamic drug clearance and defense mechanisms of the eye, reduced penetration and rapid elimination of the bulk topically administered formulations [4], resulted in the significant decrease in Senicapoc levels detectable in flushed tears over a period of 90 min in rats (Figure 7). Static barriers such as tight junctions of the corneal epithelium also dominates by preventing major penetration of drug molecules across the cornea [1,5,14]. In addition, dry eye disease is also characterized as an ocular surface disorder [1], which produces its greatest effect when drugs reside on the surface of the cornea. Hence, the quantitation of prolonged drug bioavailability in flushed tear concentration would provide the best indication of such a treatment. As a result of increased viscosity, hydrogel formulations were shown to significantly increase bioavailability, compared to a less viscous formulation of Pluronic F-127 for up to 30 min. Therapeutic dosages were also achievable within the eye for close to 60 min for hydrogel formulations, or six times longer than viscous formulations (Figure 7). This would be expected to be much longer than what is attainable with currently available free drug topical solutions. In vivo results reported by Hsiue et al. [21] have also shown that drug and drug-loaded nanoparticles encapsulated within their hydrogel increased bioavailability 5-fold over conventional eye drops, with resulting increase in efficacy. The results obtained are also consistent with the general use of hydrogels as carrier matrices to prolong and sustain therapeutic drug delivery [1]. It is also exciting to note that the gentler gradient of the release profile exhibited by liposomal hydrogel formulation (Figure 4) compared to the free liposomal formulation (Figure 3) reduces the spiking effect and contributing to a more controlled daily drug release profile.

As a consideration, sub-conjunctiva injections have also been widely explored for ocular delivery of therapeutics. This strategy is widely used to overcome the limitations posed by corneal route of penetration, for the movement of therapeutics towards the back of the eye. However, although the strategy of using sub-conjunctiva injection is mainly to target posterior eye pathologies, it has also been widely reported that drug concentrations can be detected in both anterior as well as posterior chambers of the ocular globe [4,5,10,27,28]. In particular, the article reported by Feng L. et al. [28] showed that after sub-conjunctiva injection, not only did their pRNA nanoparticles retain in the conjunctiva tissues, but the migration of their pRNA nanoparticles could be found to be up taken by the cells of the cornea and sclera. Hence, it was interesting to explore the trans-sclera route provided by sub-conjunctival injection for the treatment of ocular surface diseases. Senicapoc-loaded liposomes injected into the connective tissues within the sub-conjunctiva, can also provide a natural depot for prolonging liposomal residence by weak hydrophobic epithelial–stromal interactions [24].

Figure 8 provides insights of the residual drug concentration left within the conjunctiva tissues after 24 h, which indirectly provides correlational information to the rate of release from the liposomes, as well as trans-sclera migration of the liposomes after sub-conjunctiva injection. The possible release of drugs from the liposomes injected with the sub-conjunctiva tissues, as well as the possible migration of nanoparticles that occurs within the eye as reported by Feng L. et al. [28], would likely correlate to low detectability of Senicapoc within sub-conjuctiva tissues after 24 h (Figure 8). A limitation experienced in this sub-conjunctiva injection study was the inability to collect sufficient tears for analysis due to a combination of various factors, which includes the small volume of tear production, as well as the reduced tear production of the rats during anesthetized states. The quantification of the tear concentrations would have been useful in the comparison to tear concentration accumulated on the ocular surface of the hydrogel formulation. Another limitation of the sub-conjunctival injection experiment is that there was no time point between 1 and 24 h. While the rats were anesthetized during sub-conjunctival injection, the individual rats woke up from 1 to 4 h post-anesthesia. However, the conditions of the rat eyes were different during sleep and when awake. To compensate the possible different effects of anesthesia on individual rats, we only harvested tissue 24 h after injection. Nevertheless, articles reported by Natarajan J.V. et al. [51,52] on the sustained release of latanoprost, a therapeutic for glaucoma, characterized for the treatment of high intra-ocular pressures in the anterior chamber of rabbit eyes, from liposomal formulations showed sustained release of up to 50 days, and subsequently 90 days in the following citations respectively [51,52]. Although the results reported by Natarajan J.V. et al. [51,52] were based on in vivo efficacy studies, the results are, however, relatable and can be used as a justification of the possible migration of the nanoliposomes by trans-sclera route into the anterior chamber. This suggests a similar basis for the fabrication of Senicapoc-loaded nanoliposomes to be used in sub-conjunctiva injection, for the potential treatment of diseases of the ocular surface.

## 4. Materials and Methods

### 4.1. Materials

Senicapoc was a gift from Prof. Heike Wulff, University of California, Davis. Dipalmitoylphosphatidylcholine (DPPC) and 1,2-Dioleoyl-*sn*-Glycero-3-Phosphoethanolamine-*N*-(Carboxyfluorescein) were purchased from Avanti Polar Lipids (Abalaster, AL, USA). Whatmann drain discs and polycarbonate membrane filters were purchased from GE Healthcare Life Sciences (Freiburg, Germany). Chloroform (HPLC Grade) was purchased from Tedia Chemicals (Fairfield, OH, USA). Acetonitrile and Methanol (LCMS Grades) were purchased from Fisher Scientific (Hampton, NH, USA). Cellulose ester dialysis tubings (100 kDa MWCO) and tubing closures were obtained from Spectrum Laboratories (Rancho Dominguez, CA, USA). Salts used to make Phosphate Buffered Saline (PBS) includes sodium chloride (NaCl), potassium chloride (KCl), Di-sodium hydrogen phosphate (Na_2_HPO_4_) and Potassium dihydrogen phosphate (KH_2_PO_4_) were all purchased from Sigma-Aldrich (St. Louis, MO, USA). Zinc Sulphate Monohydrate salt for analytical processing was also purchased from Sigma-Aldrich. Soft tissue homogenizing kits (CK14, 0.5 mL) used for tissue analysis were purchased from Bertin-Instruments (Rockville, MD, USA).

### 4.2. Preparation of Liposomal Suspension

#### 4.2.1. Fluorescein-Tagged DPPC Liposomal Suspension

The 20 mM fluorescein-tagged DPPC liposomes were prepared by dissolving 11.4 mg (2 mM) of 1,2-Dioleoyl-*sn*-Glycero-3-Phosphoethanolamine-*N*-(Carboxyfluorescein) and 66.1 mg (18 mM) of DPPC lipids and chloroform: methanol with a 2:1 ratio in a round-bottom flask (RBF). The RBF was secured to a rotary evaporator and held for 60 min within a 40 °C water bath for the rapid removal of the organic solvents. A thin-layered film of the lipid mixture was eventually formed on the inner surface of the round-bottomed flask. The spontaneous formation of multilaminar vesicles (MLVs) was achieved upon rehydration with 5 mL of phosphate buffered saline (PBS, pH 7.4). The MLVs were then subjected to extrusion using a 10 mL LIPEX extruder heated at 60 °C with a circulating water bath and extruded with 5 cycles of 200 nm membrane filters followed by 10 cycles of 80 nm membrane filter to form small unilaminar vesicles (SUVs). The loss of buffer through the extrusion process was measured and reconstituted with PBS to 5 mL of final stock liposomal formulation. The stock liposomal formulation was subsequently stored at 4 °C for future use.

#### 4.2.2. Senicapoc-Loaded DPPC Liposomal Suspension

The 10 mol % Senicapoc-loaded DPPC liposomes were prepared by dissolving 3.2 mg of Senicapoc and 73.4 mg (20 mM) of DPPC lipids in chloroform: methanol with a 2:1 ratio in a round-bottomed flask (RBF). The subsequent steps for the preparation were identical to the fabrication methodology as mentioned above in Section 4.2.1.

#### 4.2.3. Pluronic F-127 Hydrogel Formulations

Senicapoc-loaded liposomes were subsequently loaded into Pluronic F-127 hydrogels by hydrating 160, 180, and 240 mg of Pluronic F-127 polymer with 1 mL of the final liposomal suspension and dissolved overnight at 4 °C to form a 16% viscous solution, 18% and 24% formulation that was capable of gelation in situ, respectively.

### 4.3. Size Measurements of Senicapoc-Loaded Liposomes

Measurement of liposome hydrodynamic size were performed using dynamic light scattering (DLS). The experiment was conducted by suspending 10 µL of Senicapoc-loaded or Fluorescein-tagged nanoliposomes in 1 mL of de-ionized (DI) water, within a cuvette. The resultant cuvette was then placed in a Malvern Zetasizer System for measurement. All measurements were made in triplicates.

### 4.4. Drug Loading of Senicapoc-Loaded Liposomal Suspension

Drug loading of formulations were indirectly quantified by back calculation for the total extruded volume of 5 mL, with a fractional volume (5 µL) measurement taken from the total extruded volume of the liposomal suspension, and subsequently quantified by liquid-liquid extraction. Quantification of fractional drug content was performed by lysing 5 µL of liposomes suspension with PBS and acetonitrile to a final ratio of 2:8 respectively. Samples were filtered through a 0.22 µm regenerated cellulose (RC) filter and subsequently analyzed using liquid chromatography mMass spectroscopy (LCMS, Waters Corporation) with multiple reaction monitoring, through a BEH 1.7 µm C-18 column, 2.1 mm × 50 mm using an in situ mixed Isocratic mobile phase of 80% Acetonitrile + 0.1% Formic Acid and 20% DI Water + 0.1% Formic Acid, at a flowrate of 0.25 mL/min. LCMS results were analyzed by comparison to a pre-developed calibration curve. Lower limit of Detection (LoD) and lower limit of Quantitation (LoQ) were determined at 0.5 ng/mL with the latter defined by signal to noise (S/N) ratio value ≥20.

### 4.5. In Vitro Stability of Senicapoc-Loaded Liposomal Suspension

Senicapoc-loaded liposomes were monitored for stability based on size and drug loading over a period of 28 days. Samples from the stock liposomal formulation stored at 4 °C were periodically considered for analysis. Characterization of liposome sizes by intensity were performed with dynamic light scattering (DLS) measurements using Malvern Nano Zetasizer. Characterization for drug loading at periodic time points were performed identically using the method as described above in Section 4.4.

### 4.6. In Vitro Release Studies of Senicapoc-Loaded Liposomes & Hydrogel Formulations

Sample volumes by mass, containing 121.5 ± 10.9 µg of Senicapoc, were dispensed into individual dialysis tubing before being secured and placed submerged within wide-mouth bottles containing 30 mL of PBS each. The bottles were subsequently placed in a 37 °C incubator with a shaking speed of 50 rpm to mimic physiological eye conditions [27]. The release assays were also performed for 28 days in triplicates to ensure consistency. Samples were aliquoted before being replaced on a daily basis with fresh PBS to maintain sink conditions. Two hundred microliters of sample was mixed thoroughly with 800 µL acetonitrile and filtered before characterizing the quantity released using the LCMS method as mentioned above.

### 4.7. In Vivo Study

#### 4.7.1. Animals

Female Sprague-Dawley rats between 6–8 weeks old (InVivos, Singapore) were used in this experiment. Animals were handled according to institutional guidelines and the ARVO Statement for the Use of Animals in Ophthalmic and Vision Research. The study protocol was approved by the Institutional Animal Care and Use Committee of SingHealth (2014/SHS/0983, approval date: 29 September 2014).

#### 4.7.2. In Vivo Residence of Pluronic F-127 Hydrogel

Three rats were anesthetized with ketamine (75 mg/kg) and xylazine (10 mg/kg). Ten microliter of fluorescein-tagged free liposome, viscous formulation (16%), and gel formulation (24%) were applied into the conjunctival sac of the rat eyes. To determine the retention time of the fluorescein-tagged liposome on ocular surface, rat eye was examined by fluorescein imaging and fluorophotometry. Fluorescein imaging on the rat ocular surface was performed with Micron IV (Phoenix Research labs, Pleasanton, CA, USA) platform with a cobalt blue filter. Rat eyes were imaged every 5 min, and to prevent dessication of rat eyes, lids were passively blinked every 30 s between two examinations.

Fluorophotometry of the rat eye was carried out with Fluorotron Master™ (OcuMetrics, Mountain View, CA, USA) with the rat lens. This equipment recorded the fluorescence signal along the visual axis in the posterior to anterior direction of the eye. Before applying the fluorescent formulation, a baseline measurement of the rat eye was obtained. After application of the formulation, the rat was held on an adjustable stand and the cornea of rat eye was adjusted to face the lens. Eye was imaged every 3 min, without any blinking between scans. This is in consideration that rat eyes are very small with an axial length of around 6 mm, hence passive blinking will change the position of the eye, and results in the inconsistent scan readings.

#### 4.7.3. In Vivo PK Analysis of Flushed Tears

The 10 µL of viscous formulation (16%) and gel formulation (24%) were applied, using standard administration procedure for topical eyedrops [1,5,10], into the conjunctival sac of the right and left eye of a rat respectively. The rat eyes were passively blinked every 30 s to prevent desiccation of the ocular surface. Eye-flush tears were collected at 4 specific time points after 10, 30, 60 and 90 min. At each time point, the 10 µL of PBS was instilled on rat cornea, the rat eye was passively blinked 3 times, and then 10 µL of flush tears collected by a 10-µL capillary tube (Sigma-Aldrich, St. Louis, MO, USA) and stored under −80 °C. Baseline eye-flush tears collected before formulation instillation was used as control. Senicapoc in tear samples were quantified by mixing 1 µL of the tear sample with specific volumes of PBS and acetonitrile to achieve a final ratio of 1:4 and subsequently analyzed using LCMS method as described in Section 4.4.

#### 4.7.4. In Vivo PK Analysis of Sub-Conjunctival Injection

The 18 rats were anesthetized prior to the injection of Senicapoc nanoliposome (5 µL) into the subconjunctiva of right eye. Left eye were injected with the same volume of PBS as control. Subconjunctiva injection was performed by gently pulling conjunctiva from the sclera with a pair of forceps, and nanoliposome or PBS was injected into the superior subconjunctival region using a 50 µL Hamilton syringe with a 30G needle. Injected rats were randomly divided into 3 groups according to the study plan as shown in Table 1.

Conjunctival tissues covering the eye globe from limbus to fornix were harvested from both eyes at 1 h, 24 h and 3 weeks after injection. Harvested tissues were stored at −80 °C until use. Conjunctival tissues from another 3 control rats were used for LC-MS baseline and calibration. Senicapoc in conjunctival tissues were extracted after tissue homogenization with PBS and acetonitrile in a ratio of 1:4 at 10,000 rpm on 30 s “on” followed by 30 s “off” Interval for 2 cycles, before quantification by the LC-MS.

### 4.8. Statistical Analysis

Results are reported as mean ± standard deviation. Parametric two-tailed independent *t*-test analysis were used to compare means between experimental groups and a *p-*value of <0.05 was considered statistically significant.

## 5. Conclusions

In this study, we discuss two potential strategies to administer our novel formulations of Senicapoc-loaded hydrogel as a topical eye drop, and Senicapoc-loaded liposomes as a sub-conjunctiva injectable, as treatment strategies for the targeting of ocular surface diseases. Senicapoc-loaded liposomes were prepared in a thermo-sensitive hydrogel matrix of Pluronic F-127. In vitro results show that Senicapoc can be released sustainably from selected DPPC liposomes over an extended period of 28 days. In vivo studies show that Pluronic F-127 hydrogel at 24 wt% concentration increases the residence time of the nanoliposomes on the surface of the eye, increases bioavailability, and also supports the penetration of the nanoliposomes into the eye. Alternatively, Senicapoc can be administered via sub-conjunctival injection, the tissues PK remains for up to 24 h post-injection.

## Figures and Tables

**Table 1:** Study Plan for in vivo PK study of Sub-conjunctiva Injection.

Study Plan								
Sample Formulation	8.5 mol % Senicapoc-Loaded Liposomal Formulation							
Timepoints	Baseline/Calibration		1 h		24 h		3 weeks	
Number of Rats	3		6		6		6	
Selection of Eye	Left	Right	Left	Right	Left	Right	Left	Right
Type of Eye Drop	-	-	PBS Control	Sample	PBS Control	Sample	PBS Control	Sample
